# The role of diabetic foot treatment in improving left ventricular function: Insights from global longitudinal strain echocardiography

**DOI:** 10.1371/journal.pone.0299887

**Published:** 2024-03-29

**Authors:** Mohammad Taghi Ashoobi, Hosein Hemmati, Maziar Moayerifar, Mani Moayerifar, Mahboobeh Gholipour, Mahsa Motiei, Mohammad Ali Yazdanipour, Habib Eslami Kenarsari

**Affiliations:** 1 Department of Vascular Surgery, Razi Clinical Research Development Unit, Razi Hospital, Guilan University of Medical Sciences, Rasht, Iran; 2 Department of Cardiology, Healthy Heart Research Center, Heshmat Hospital, School of Medicine, Guilan University of Medical Sciences, Rasht, Iran; 3 School of Medicine, Razi Hospital, Guilan University of Medical Sciences, Rasht, Iran; 4 Neuroscience Research Center, Trauma Institute, Guilan University of Medical Sciences, Rasht, Iran; 5 Vice-Chancellorship of Research and Technology, Guilan University of Medical Science, Rasht, Iran; Isawiya General Hospital, Governorate of Gurayyat, SAUDI ARABIA

## Abstract

We decided to evaluate the effect of treatment of diabetic foot ulcers in improving heart function by strain echocardiography than conventional transthoracic echocardiography. This prospective cross-sectional study included patients with diabetic foot ulcer (DFU). Conventional and two-dimensional strain echocardiography performed before and after three months diabetic foot treatment. Then, we compared the echocardiographic parameters including left ventricular ejection fraction (LV-EF), left ventricular global longitudinal strain (LV-GLS). Multivariate and univariate logistic regression analysis were performed to find which variable was mainly associated with LV-GLS changes. 62 patients with DFU were conducted. After echocardiography, all patients underwent surgical or non-surgical treatments. Three months after the treatment, LV-EF was not significantly different with its’ primary values (P = 0.250), but LV-GLS became significantly different (P<0.05). In the multivariate logistic regression analysis, with the increase in the grade of ulcer, LV-GLS improved by 6.3 times. Not only the treatment of DFU helps to control adverse outcomes like infection, limb loss and morbidity but also it enhances cardiac function. Of note, strain echocardiography found to be a better indicator of myocardial dysfunction than LV-EF. These findings make a strong reason for the routine assessment of cardiac function in patients with DFU.

## Introduction

Diabetes mellitus (DM) associates with decreased insulin secretion from pancreas, which results in elevated blood glucose. Globally, one in every 11 adults involves with Diabetes mellitus(DM) [1]. Diabetic foot ulcer (DFU) as one of the most prevalent and debilitating complications of Diabetic mellitus, mainly results from peripheral vascular disease or neuropathy, and occurs in 15 to 25% of these patients during their lifetime [2]. Heart failure develops in diabetic patients over twice higher than non-diabetics [3]. Impaired microvascular endothelial function and altered metabolism of glucose, followed by free fatty acids oxidation in myocardium, can be the cause of systolic and diastolic dysfunction in diabetics [3].

Early detection of heart failure offers the best chance of cure in these patients. Nowadays, the most common method for detecting heart failure is measuring left ventricle ejection fraction (LV-EF), by echocardiography. Despite the easy application of LV-EF, it remains normal in early stages of cardiac disease and it’s considered as heart failure with preserved LVEF (HFpEF). We hope that, Strain monitoring as a new method enables us to detect the early minimal changes in myocardium.

Based on a recent study, DFU treatment improves cardiac function assessed by strain measurements [4]. At present, few data is available about the ability of strain echocardiography in prediction of systolic dysfunction in DFU, and the possible effect of the treatment of these patients on their cardiac function. So, we decided to evaluate the effect of treatment of diabetic foot ulcers in improving heart function by this new and efficient method-strain echocardiography- which provides a better estimate of the diagnosis and staging of heart disease than LV-EF.

## Materials and methods

### Study design

Participants were prospectively evaluated (29 July 2022–30 April 2023) in this analytic cross-sectional study, approved by the local ethics committee of Guilan University of Medical Sciences (Code: IR.GUMS.REC.1401.241). The study was conducted in accordance with the ethical standards laid down in the 1964 Declaration of Helsinki and its later amendments. Written informed consent was obtained from all participants.

### Participants

Diabetic Patients with DFU, who were diagnosed by a vascular surgeon based on Diabetic Foot Study Group guidelines, included in this study. Diabetic Patients were who used anti-diabetic oral medication, insulin or who had fasting blood glucose (FBS) ≥ 126 mg/dl for at least two times. After echocardiographic evaluation, all the patients underwent therapeutic interventions, in our diabetic foot center, supervised by a vascular specialist. Patients with acute or advanced stage of renal, hepatic or pulmonary diseases, acute coronary syndrome, known coronary artery disease in myocardial perfusion imaging and coronary CT angiography, malignancy, infectious disease in the last two weeks, coagulopathy, history of a hemorrhagic stroke, severe valvular disease and heart failure (EF<45%) were excluded.

We recorded any concomitant diseases or any history of coronary artery disease, ischemic heart disease and excluded all patients with more than 50 percent stenosis in coronary arteries based on CT angiography. Or cerebral stroke. A physical examination was performed for each patient. We measured participants’ height and weight and calculated body mass index (BMI) and body surface area (BSA). A vascular specialist examined their ulcer to note its grade based on Wagner-Megitt classification. After 12 hours of fasting a blood sample was taken from patients to measure the level of total cholesterol, triglyceride, low-density lipoprotein (LDL) and high-density lipoprotein. Hyperlipidemia was defined as LDL>130 mg/dl or use of cholesterol-lowering drug. Smokers were participants with a history of smoking for 12 months, regularly.

### Echocardiography

A cardiologist performed echocardiography using Samsung HS70A Ultrasound Machine on all participants. Each parameter was tested three times in sinus rhythm, and the mean value was noted. Left ventricular (LV) volumes and LV-EF assessed by modified biplane Simpson’s method using M-mode and Two-dimensional (2D) images from the apical four- and two-chamber views at 2.0 to 3.5 MHz. And divided to two categories: normal >55%, mild LV systolic dysfunction 45–55%. LA volume in end systole assessed by the biplane area-length method, from apical 4- and 2-chamber views and then indexed by BSA to have LAVI. At the end of ventricular systole, the largest volume of RA was measured from the lateral aspect of the tricuspid annulus to the septal aspect (the region between the leaflets and annulus was excluded). Transmitral LV inflow in an apical 4-chamber view was used to measure E and A waves and E/A ratio during atrial systole was measured as well as early diastolic velocities (e’) at the septal and lateral mitral annulus from apical 4-chamber view. Peak TR velocity determined by the right ventricular inflow measurement with the maximum value in parasternal short-axis from apical 4-chamber views. Diastolic dysfunction determined by using the algorithms from the ASE guidelines.

### LV strain echocardiography

**Fig 1:**
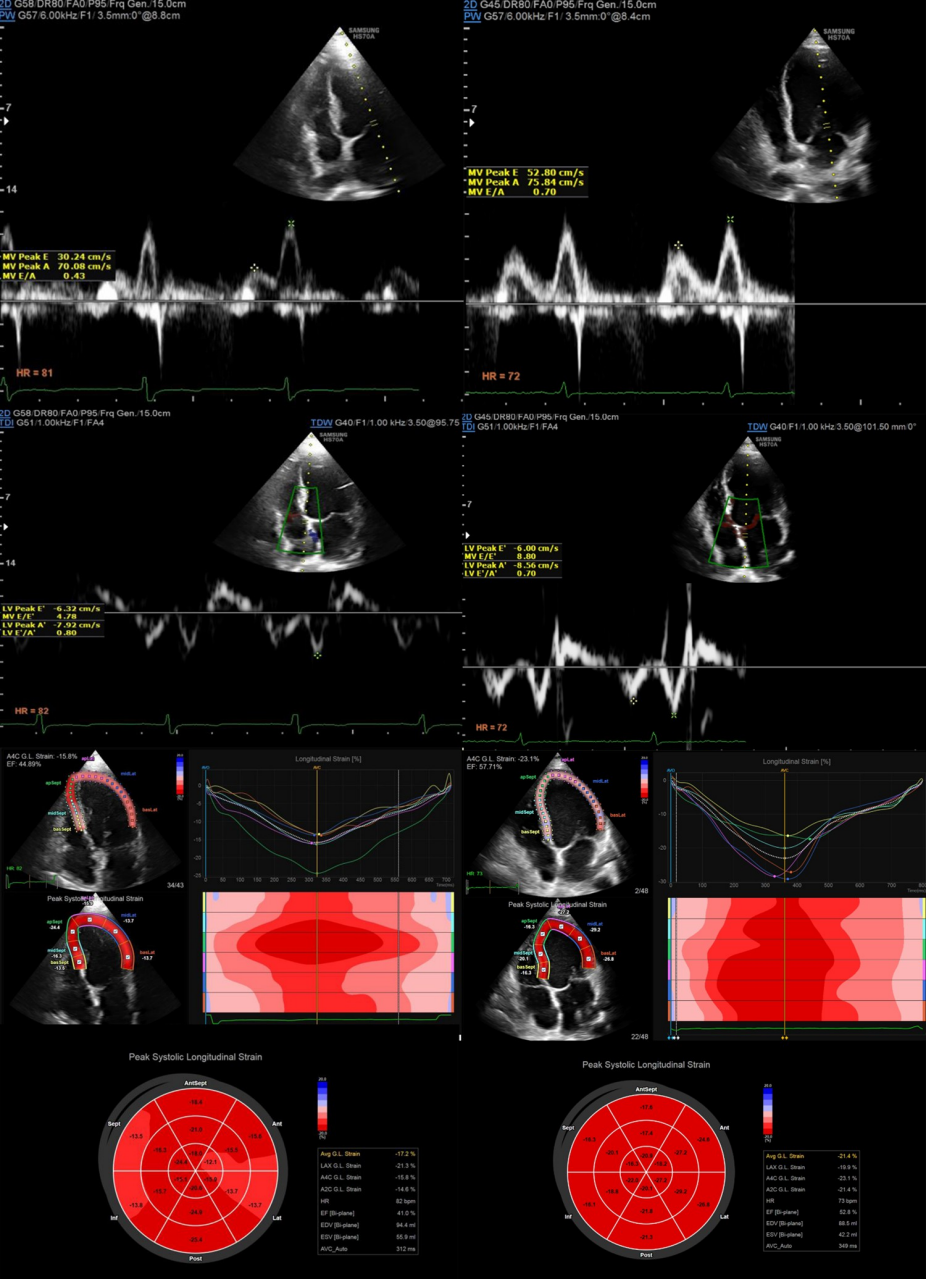
Displays doppler and strain echocardiographic images of patients with DFU before treatment on the left side and after treatment on the right side in order from top to bottom. Firstly, there is a pulsed wave Doppler recording at the miral inflow E and A velocities. secondly, tissue Doppler imaging at the medial mitral annulus shows e’ and a’ velocities. Thirdly, a region of interest tracing in the apical 4-chamber views shows segments of left ventricle and peak systolic strain waveform in mid-panel. Fourthly, bull’s eye plot of longitudinal strain is shown, diplaying strain values of each segment.

LV strain echocardiography was carried out using a Samsung HS70A ultrasound Machine (version: 2.01.04.0528) with a modified frame rate of 60 to 80 Hz, when patients were lying in the left lateral decubitus posture with attached electrocardiographic electrodes. Strain rate analysis was done. The cardiologist traced the endocardium manually by the beginning of systole and taking images from apical views (A2B, A3B, A4B). Then, longitudinal strain (GLS) measurement performed by the average value of the 18. LV segments divided by the analytical software when acoustic tracking software tracked myocardial speckle pattern and categorized in 3 groups (Fig 1). 10–15% increase in LV-GLS after treatment was considered as improvement.

### Diabetic foot diagnosis and treatment

All the patients attending to outpatient underwent comprehensive physical examination and Para clinic interventions. Examinations performed by a vascular surgeon to note any neuropathic or vascular involvement. The physicians also examined the depth and with of ulcer or any gangrene based on Wagner-Megitt classification. X-ray Radiography images also obtained of involved foot to notice if there is osteomyelitis or not. After then, Patients hospitalized in our hospital to perform ankle-brachial index (ABI) and toe-brachial index (TBI) measurements and initiate therapeutic interventions. Non-surgical therapies including antibiotic therapy, tight glycemic control, in-office debridement, frequent wound dressings and endovascular treatment as well as surgical therapies including radical debridement, grafts and amputations performed. Whenever needed, we consulted with infectious disease specialist, endocrinologist, and orthopedist in order to achieve the most desirable treatment goal.

### Statistical analysis

Statistical analysis was performed using the R-software v.4.1.2 [The R Foundation for Statistical Computing, Vienna, Austria] with “Hmisc”, “stats”, “psych and “foreign” packages. Continuous variables were expressed in mean ± standard deviation (SD), while categorical variables were expressed in number and percentage. The Echocardiography findings before and after diabetic foot treatment were compared using paired samples t-test and Wilcoxon Signed Rank test. Univariate analysis was carried out to identify the factors associated with improvement in LV-GLS. Factors with a p-value < 0.1 in the univariate analysis were included in multivariable logistic regression analyses to assess the impact of demographic, clinical data, and ulcer index variables on the improvement of LV-GLS in patients with diabetic foot. All P-value for the tests were two-sided, and P-value <0.05 were deemed statistically significant.

## Results

Of the 85 patients with DFU enrolled in this study, 5 patients (5.8%) died during the follow-up period, 18 (21.1%) patients were excluded from all subsequent analyses because they were unwilling to participate in study and perform control echocardiography after receiving therapy for their diabetic foot. As a result, in 62 of 85 (72.9%) patients obtained three months’ follow-up data. Participants had a mean age of 61.6 ± 7.9 (range, 46–78) years. Hypertension (n = 58, 93.5%) and hyperlipidemia (n = 57, 91.9%) were the most prevalent underlying diseases in this sample (Table 1).

**Table 1:** Demographic, and clinical data of patients with diabetic foot (n = 62).

Age, years	Mean(SD)		61.6(7.9)
Gender	N (%)	Female	29(46.8)
		Male	33(53.2)
BMI, kg / m^2^	Mean(SD)		24.9(2.6)
	N (%)	Normal	30(48.4)
	N (%)	Overweight	32(51.6)
Smoke	N (%)		26(41.9)
Hypertension	N (%)		58(93.5)
Hyperlipidemia	N (%)		57(91.9)
CAD	N (%)		25(40.3)
CVA	N (%)		15(24.2)
COPD	N (%)		8(12.9)

BMI: body mass index, CAD = coronary artery disease, CVA = cerebrovascular accident, COPD = chronic obstructive pulmonary disease

The ABI of diabetic subjects ranged from an ischemic value of 0.7 to a clearly calcified level of 1.6 with a mean value of 0.9 ± 0.1. Abnormal ABI and TBI values were found in 61.3% (n = 38) and 88.7% (n = 55) patients, and the majority of patients had DFU with grade 2 and 3 (40.3% and 35.5%), respectively. Amputation and radical debridement were performed on 28(45.2%) and 12(19.3%) patients, and others had endovascular, new dressings and graft treatment procedures (Table 2).

**Table 2:** Ulcer indexes, and treatment data of patients with diabetic foot (n = 62).

ABI	N (%)	Normal>0.9	24(38.7)
		Abnormal< = 0.9	38(61.3)
TBI	N (%)	Normal>0.7	7(11.3)
		Abnormal< = 0.7	55(88.7)
Toe pressure	N (%)	<55	24(38.7)
		> = 55	38(61.3)
**DFU degrees (Wagner classification)**	N (%)	0	1(1.6)
		1	10(16.1)
		2	25(40.3)
		3	22(35.5)
		4	4(6.5)
		5	0(0.0)
Treatment Type	N (%)	Surgical	41(66.1)
		Non-surgical	21(33.9)

ABI: ankle brachial index, TBI: toe brachial index.

Echocardiographic assessment of this population by using Paired Samples T-Test and Wilcoxon Signed Ranks Test showed that all primary echocardiographic parameters except LVEF became significantly different at the 3rd-month follow-up (Table 3).

**Table 3:** Comparison of echocardiography findings of patients with diabetic foot before and after treatment (n = 62).

		Before	After	P-Value
		N (%)	N (%)	
	Mean(SD)	50.1(4.7)	50.6(4.3)	0.041^†^
LV-EF	Normal (>55%)	43(69.4)	46(74.2)	0.250^‡^
	Mild (45–55%)	19(30.6)	16(25.8)	
	Mean(SD)	-16.6(2.9)	-20.6(2.3)	<0.001^†^
LV-GLS	Normal (≥18)	12(19.4)	58(93.6)	<0.001^‡^
	border line (16–18)	19(30.6)	3(4.8)	
	Abnormal (<16)	31(50.0)	1(1.6)	
	Mean(SD)	28.3(6.0)	25.7(5.0)	<0.001^†^
LAVI	16–33	46(74.2)	58(93.5)	<0.001^‡^
	34–41	16(25.8)	4(6.5)	
	Mean(SD)	14.4(2.5)	13.1(2.1)	<0.001^†^
RAVI	<18	49(79.0)	59(95.2)	0.002^‡^
	> = 18	13(21.0)	3(4.8)	
e’ septal		6.7(1.6)	7.5(1.2)	<0.001^†^
e’ lateral		9.1(1.5)	9.9(1.5)	<0.001^†^
a’ septal		8.5(1.6)	9.5(1.6)	<0.001^†^
a’ lateral		11.0(1.7)	12.1(1.9)	<0.001^†^
E		62.7(15.6)	66.0(13.9)	<0.001^†^
A		70.6(18.1)	77.4(17.7)	<0.001^†^
Pulmonary atrial pressure		26.8(4.3)	23.8(3.4)	<0.001^†^
E/A		0.9(0.2)	0.8(0.1)	0.037^†^
E/e’		9.7(3.0)	8.8(2.3)	<0.001^†^
Diastolic dysfunction	mild	40(64.5)	59(95.2)	<0.001^‡^
	moderate	22(35.5)	3(4.8)	

LV-EF: left ventricular ejection fraction, LV-GLS: left ventricle global longitudinal strain, LAVI = left atrial volume index, RAVI = right atrial volume index

^**†**^ paired samples t-test

^‡^Wilcoxon Signed Rank test

Univariate and multivariate logistic regression used for relationship between LV-GLS improvement and individual and clinical factors. LV-GLS increased in the univariate analysis with age and the grade of diabetic foot ulcer, but decreased by increasing BMI. So that the chance of improvement of LV-GLS after diabetic foot treatment increased by 9% with the increase of one year of age (OR = 1.09, P = 0.046). Moreover, with an increase of one degree of diabetic foot ulcer, the chance of improving LV-GLS was6.3 times (OR = 6.32, P = 0.001). LV-GLS improvement decreased 23% by one unit increasing of BMI (OR = 0.67, P = 0.014). However, in the multivariate logistic regression analysis, only the grade of diabetic foot ulcer was related to the improvement of LV-GLS by 4.5 times (OR = 4.54, P = 0.009) (Table 4).

**Table 4:** Association LV-GLS improved with demographic, clinical data, ulcer indexes, and treatment data of patients with diabetic foot by logistic regression.

	univariate		multivariate	
	OR(CI 95% OR)	P-Value	OR(CI 95% OR)	P-Value
Age	1.09(1.00–1.18)	0.046	1.02(0.92–1.12)	0.759
Gender(male/female)	1.71(0.51–5.70)	0.380		
BMI	0.67(0.48–0.92)	0.014	0.77(0.53–1.14)	0.193
Smoke(yes/NO)	1.40(0.41–4.80)	0.593		
Hypertension (yes/NO)	1.15(1.11–12.05)	0.905		
Hyperlipidemia (yes/NO)	2.50(0.37–16.70)	0.344		
CAD (yes/NO)	3.10(0.77–12.55)	0.112		
CVA (yes/NO)	5.35(0.64–44.91)	0.122		
ABI (abnormal/normal)	1.82(0.55–6.07)	0.328		
Toe pressure(<55/> = 55)	2.85(0.70–11.54)	0.142		
DFU grades	6.32(2.17–18.42)	0.001	4.54(1.47–13.99)	0.009
Treatment Type (Surgical/ Non-surgical)	1.11(0.32–3.87)	0.868		

BMI: body mass index, CAD: coronary artery disease, CVA: cerebrovascular accident, ABI: ankle-brachial index, DFU: diabetic foot ulcer

## Discussion

The most important finding of this study is that treating diabetic foot ulcers in patients who suffer from this condition can lead to an improvement in cardiac function, based on strain echocardiographic features. Heart diseases are the leading cause of morbidity and mortality in diabetic patients and are known to cause about two to six times more mortality in these patients than individuals without diabetes [5]. Patients with DFU syndrome are even at higher risk of developing cardiovascular diseases than diabetic patients without DFU. These patients usually have underlying metabolic disorders like hypertriglyceridemia and hyperglycemia which cause microvascular injury over time [6, 7]. The population of DFU patients in current study had also high prevalence of hypertension and hyperlipidemia as cardiovascular risk factors. Patients with DFU may too present diabetic peripheral neuropathy which increases the risk of silent myocardial ischemia development [8]. Despite this, diabetic foot centers mainly focus on foot care than cardiac assessment. Medical history, physical examination and blood tests as first line diagnostic tools do not provide adequate information about cardiac status, such as diastolic or systolic function, left ventricular hypertrophy and silent myocardial ischemia which can be precisely diagnosed by using echocardiographic parameters [9]. This issue highlights the importance of cardiac assessment and echocardiographic examination in diabetic patients, especially those with DFU.

In this study, we observed a significant difference in both atrial volume indices before and after treatment. Diabetes affects atrium in several ways [10]. Diastolic dysfunction of left ventricle, a frequent finding in diabetic patients, may cause impaired left atrial phasic function [11]. Diabetic patients may also develop sub endocardial fibrosis, impaired autonomic nervous system, high blood pressure and systemic inflammation which can induce decreased wall elasticity and changes in atrial anatomy and function [12, 13]. Changes in the volume of atrium with the treatment of diabetic foot is a new and interesting finding of current study, and more studies are needed to validate this association.

Strain echocardiography, as a new popular method, evaluates myocardial function by measuring longitudinal myocardial shortening which it not visually detectable, so it helps cardiologists to detect systolic dysfunction sooner [14]. Recent studies have shown widely usage of strain echocardiography in detecting cardiotoxicity of cancer patients [15] as well as inflammatory and infiltrative diseases like arthritis rheumatoid, Behcet’s disease and amyloidosis [16–18]. It has been also used in diabetic patients to evaluate diabetic cardiomyopathy in some studies [19, 20]. According to higher risk of cardiovascular problems in patients with DFU, early detection of cardiac dysfunction in this population was a major concern for us. Because there have been few studies evaluating the use of strain echocardiography in these patients we aimed to conduct a thorough investigation in this regard. A recent study in this field by Demirtas et al. supported our research and showed that systolic dysfunction was higher in patients with DFU, and a three-months follow up after foot ulcer treatment showed that although LV-EF was not significantly different at the end of follow-up, LV-GLS values significantly improved, but in our study we excluded obstructive coronary artery disease that influence on LV-GLS. Also we compared LV- GLS in three groups and categorized all patients in two treatment groups and evaluated each treatment group effect on LV-GLS improvement. Furthermore we found that diabetic foot ulcer (DFU) degree affected on LV-GLS improvement [4].

Before treatment, in our population 43% of patients had normal systolic function and 19% had mild systolic dysfunction, based on LV-EF values. In contrast, in the study by Löndahl et al., 78% of patients with diabetic foot ulcers had left ventricular dysfunction [9]. Indeed, in this study assessment of LV-GLS values showed that 31% of our population had systolic dysfunction before treatment, which is in line with several previous studies that has shown that GLS can detect systolic dysfunction before LVEF declines. LV-GLS has been described to be a superior to LV-EF for prediction of left ventricular systolic dysfunction [21–23].

We found that with one-unit increase in BMI, the chance of LV-GLS improvement after diabetic foot therapy dropped by 33%. Diabetes and BMI have significant effects on LV-GLS [24], and patients with higher BMI may develop cardiac steatosis and interstitial fibrosis which are associated with impaired LV-GLS [25, 26]. The results of a study by Dong et al. showed that patients with higher BMI values and those with diabetes had increased cardiac steatosis and interstitial fibrosis, and both of these factors were determinants of impaired LV-GLS [27]. The negative effect of increased BMI and diabetes on myocardial systolic function was also expressed in a multicenter study by Arnold et al. [24].

we investigated more aspects of LV-GLS and its role in assessment of cardiac function and its’ improvement after treatment. Ulcer severity was the only variable significantly associated with GLS improvement in multivariate logistic regression analysis, but there was no significant association between the type of treatment and GLS improvement. This issue demonstrates that regardless of treatment type, whether surgical or non-surgical, patients with diabetic foot may fortunately get advantage from their ulcer healing. However, we cannot ignore the role of proper diagnosis of the physicians and expert vascular surgeons who cooperated with us in the treatment of our patients’ foot ulcer. A study published in 2016 examined the relationship between foot ulcer severity and cardiac autonomic system in patents with DFU. The study found that the severity of foot ulcer was significantly associated with cardiac autonomic neuropathy [28]. Moreover, echocardiographic findings have been suggested to be indicators of long term cardiovascular outcome after vascular surgeries. A study by Choi et al. showed that preoperative LV-EF predicts the mortality rate after foot amputation [29]. These data suggest that correlation between the severity of foot ulcers and cardiac dysfunction. However, based on our knowledge there is no other study about the effect of ulcer treatment on the status of cardiac function. Thus, this is the first study to examine the effect of DFU’s treatment on myocardial function. To obtain more specific information on the relationship between foot ulcer severity and cardiac function, it is recommended that in future studies diabetic foot patients undergo comprehensive cardiovascular assessment based on individual demographic, laboratory parameters, duration of diabetes and other cardiovascular risk factors.

## Limitations

This study had also some limitations. The sample size of current study is small, and it may not have enough statistical powers to detect more significant associations. In this case, a larger sample size in future studies is needed to confirm the results. We did not have patients with moderate or severe systolic dysfunction based on LV-EF values, and our results cannot be attribute to these patients. A three-months follow-up period in this study may not have allowed for the long term effects of diabetic foot treatment on left ventricular function to be fully evaluated. Also, we conducted a wide range of age in this study and believe that older age influence on Strain echocardiography and suggest to evaluate an specific range of age. Some patients didn’t understand the effect of strain echocardiography on diabetic foot treatment and for this reason didn’t take part for follow up echocardiography. Patients in our study compare before and after treatment, so it was better if there is a control group without diabetic foot for comparison.

## Conclusions

Early detection and management of cardiac conditions are crucial to improve outcomes and reduce cardiovascular events in diabetic foot patients. The key message of our study is the use of global longitudinal strain echocardiography as a diagnostic tool allows for a more accurate assessment of myocardial function. This non-invasive technique provides detailed information on myocardial deformation, facilitating the identification of subtle changes in cardiac performance. LV-GLS is able to identify subclinical LV dysfunction earlier than LVEF measurement in patients with diabetic foot and lower in severe wound scale and use to guide early therapy both for diabetic foot and cardio-protective treatment.

## Supporting information

S1 Checklist Human participants research checklist. (DOCX)

S1 Data (XLSX)
